# Ultra-Sensitive Sequencing Reveals an Age-Related Increase in Somatic Mitochondrial Mutations That Are Inconsistent with Oxidative Damage

**DOI:** 10.1371/journal.pgen.1003794

**Published:** 2013-09-26

**Authors:** Scott R. Kennedy, Jesse J. Salk, Michael W. Schmitt, Lawrence A. Loeb

**Affiliations:** 1 Department of Pathology, University of Washington, Seattle, Washington, United States of America; 2 Department of Medicine, University of Washington, Seattle, Washington, United States of America; 3 Department of Biochemistry, University of Washington, Seattle, Washington, United States of America; University of Pittsburgh, United States of America

## Abstract

Mitochondrial DNA (mtDNA) is believed to be highly vulnerable to age-associated damage and mutagenesis by reactive oxygen species (ROS). However, somatic mtDNA mutations have historically been difficult to study because of technical limitations in accurately quantifying rare mtDNA mutations. We have applied the highly sensitive Duplex Sequencing methodology, which can detect a single mutation among >10^7^ wild type molecules, to sequence mtDNA purified from human brain tissue from both young and old individuals with unprecedented accuracy. We find that the frequency of point mutations increases ∼5-fold over the course of 80 years of life. Overall, the mutation spectra of both groups are comprised predominantly of transition mutations, consistent with misincorporation by DNA polymerase γ or deamination of cytidine and adenosine as the primary mutagenic events in mtDNA. Surprisingly, G→T mutations, considered the hallmark of oxidative damage to DNA, do not significantly increase with age. We observe a non-uniform, age-independent distribution of mutations in mtDNA, with the D-loop exhibiting a significantly higher mutation frequency than the rest of the genome. The coding regions, but not the D-loop, exhibit a pronounced asymmetric accumulation of mutations between the two strands, with G→A and T→C mutations occurring more often on the light strand than the heavy strand. The patterns and biases we observe in our data closely mirror the mutational spectrum which has been reported in studies of human populations and closely related species. Overall our results argue against oxidative damage being a major driver of aging and suggest that replication errors by DNA polymerase γ and/or spontaneous base hydrolysis are responsible for the bulk of accumulating point mutations in mtDNA.

## Author Summary

Owing to their evolutionary history, mitochondria harbor independently replicating genomes. Failure to faithfully transmit the genetic information of mtDNA during replication can lead to the production of dysfunctional electron transport proteins and a subsequent decline in energy production. Cellularly-derived reactive oxygen species (ROS) and environmental agents preferentially damage mtDNA compared to nuclear DNA. However, little is known about the consequences of mtDNA damage for mutagenesis. This lack of knowledge stems, in part, from an absence of methods capable of accurately detecting these mutations throughout the mitochondrial genome. Using a new, highly sensitive DNA sequencing strategy, we find that the frequency of point mutations is 10–100-fold lower than what has been previously reported using less precise means. Moreover, the frequency increases 5-fold over an 80 year lifespan. We also find that it is predominantly transition mutations, rather than mutations commonly associated with oxidative damage to mtDNA, that increase with age. This finding is inconsistent with free radical theories of aging. Finally, the mutagenic patterns and biases we observe in our data are similar to what is seen in population studies of mitochondrial polymorphisms and suggest a common mechanism by which somatic and germline mtDNA mutations arise.

## Introduction

Mitochondria are the primary source of energy for cells. Owing to their evolutionary history, these organelles harbor a small, independently replicated genome (mtDNA). Human mtDNA encodes two rRNA genes, 13 protein coding genes that are essential components of the electron transport chain (ETC), and a full complement of 22 tRNAs used in translation of the ETC peptides. The escape of electrons from the ETC can lead to the formation of reactive oxygen species (ROS), which are capable of damaging a variety of cellular components, including DNA. Due to its proximity to the ETC, absence of protective histones, and a lack of nucleotide excision or mismatch repair, mtDNA is thought to be especially vulnerable to ROS-mediated damage and the generation of mutations. Failure to faithfully transmit the encoded information during mtDNA replication leads to the production of dysfunctional ETC proteins, leading to the release of more free electrons and ROS in what has been termed ‘the vicious cycle’ [1], [2]. Thus, it is not surprising that mutations in mtDNA have been associated with a decline in energy production, a loss of organismal fitness, an increased propensity for a number of pathological conditions, and aging (reviewed in [3], [4]).

Numerous lines of evidence have suggested mtDNA mutations are involved in the aging process. In particular, ETC activity declines with age [5], [6], and this decrease is coincident with accumulation of mitochondria with large deletions in their mtDNA [7], [8], [9], [10]. Large, kilobase-sized deletions in mtDNA become more prevalent with age in a variety of tissues, including brain [11], heart [12], and skeletal muscle [7]. Furthermore, these large deletions have been shown to increase in frequency in a number of neurodegenerative conditions, including Parkinson's disease [13], [14] and Alzheimer's disease [15]. In addition, DNA damage, predominantly in the form of 8-hydroxy-2′-deoxyguanosine (8-oxo-dG) [16], increases with age in both nuclear and mitochondrial DNA [17], [18], [19], [20]. While the role of mtDNA deletions in aging is well established, the role of point mutations remains controversial [21], [22].

Several previous studies have examined the accumulation of point mutations in human aging and disease [23], [24], [25], [26]. Until very recently, hypotheses that required the observation of rare mutations in mtDNA have been extremely difficult to experimentally validate due to: 1) the lack of genetic tools for introducing reporters or selectable markers into mtDNA; 2) the high background error rate of most DNA sequencing methods [27], [28]; and 3) the sampling limitations of the few available high-sensitivity mutation assays that screen only a tiny subset of the genome [29]. The mitochondrial genome is 16,569 bp, and individual human cells frequently contain hundreds to thousands of molecules of mtDNA; thus, a single human cell typically contains millions of nucleotides of mtDNA sequence. The rate of accumulation of mtDNA mutations has previously been estimated as 6×10^−8^ mutations per base pair per year [30]. Therefore, reliable study of spontaneous mtDNA mutations requires methodologies that can accurately detect a single mutation among >10^6^ wild-type base-pairs. However, most prior studies of mtDNA mutations and aging have relied upon methods with background error frequencies of 10^−3^ to 10^−4^; hence the many reported differences likely reflect changes in mutation clonality or technical artifacts (e.g. due to increases in DNA damage with age) rather than true spontaneous mutations.

Massively parallel sequencing technologies allow mtDNA to be subjected to ‘deep sequencing’ in order to detect rare/sub-clonal mutations on a genome-wide level. However, these new sequencing methods are highly error prone, with artifactual error rates of approximately one spurious mutation per 100 to 1,000 nucleotides sequenced. These high error rates have precluded the study of spontaneous mutations in mtDNA [31]. To circumvent this limitation, we recently developed a new, highly accurate sequencing methodology termed Duplex Sequencing (DS), which has the unique ability to detect a single mutation among >10^7^ sequenced bases [32].

In the study herein, we determined the effect of aging on mtDNA mutation burden by using DS to compare human mtDNA purified from brain tissue of five young individuals (ages <1) and five aged individuals (ages 75–99 years) obtained via rapid autopsy (Table S1). As brain is among the most metabolically active tissues in the human body, we reasoned it to be particularly prone to damage from ROS, and thus, an optimal tissue for comparison between age groups. We assessed the relative frequency, spectrum, and distribution of mtDNA mutations in the two groups. We find that point mutations increase with age, but do so in a non-uniform manner. Furthermore, we find that mutations show a bias in their occurrence with respect to both genome location and strand orientation. The types of mutations we detect are inconsistent with oxidative damage being a major driver of mtDNA mutagenesis.

## Results

**Figure 1:**
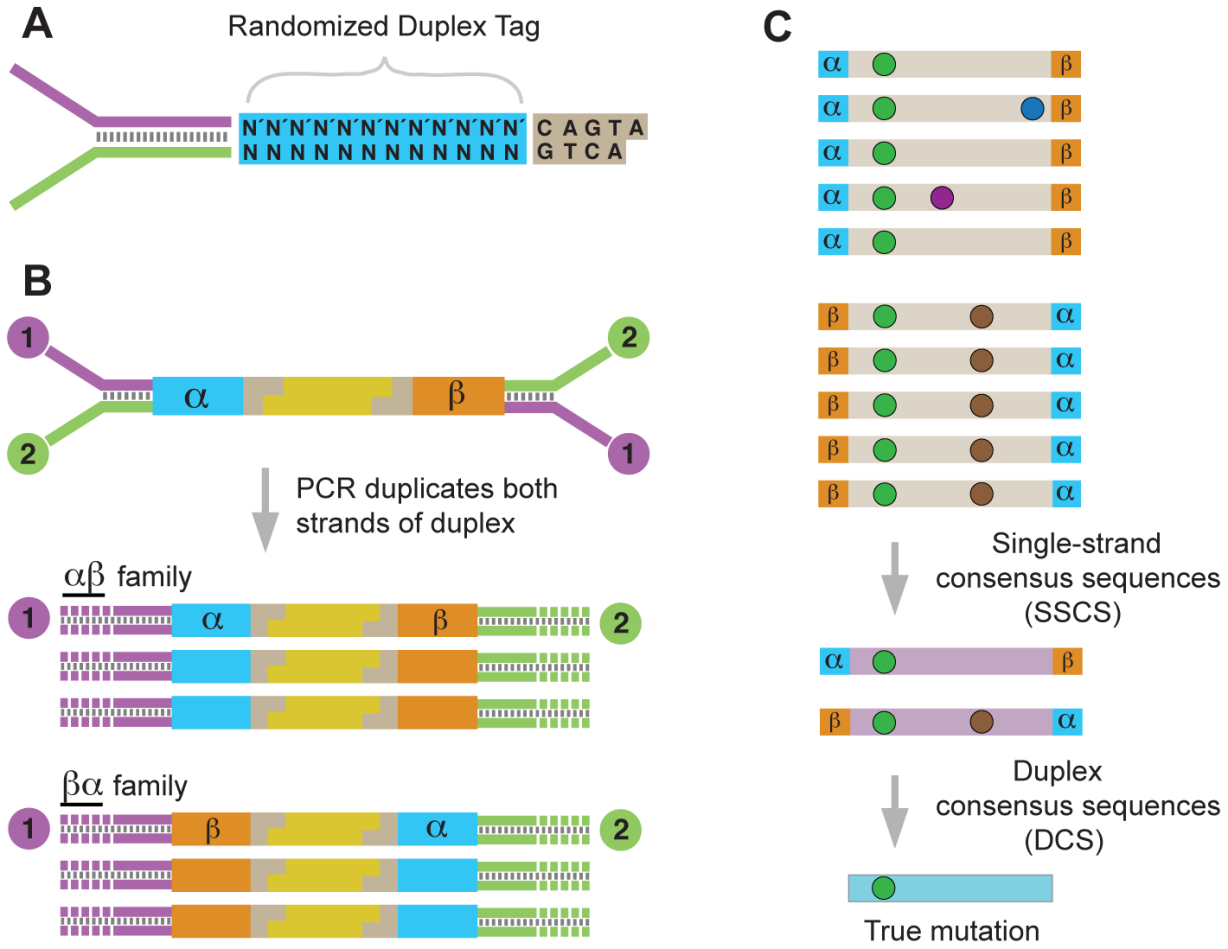
Overview of the Duplex Sequencing methodology. (A) Adapter design with random double-stranded tag sequence and invariant spacer sequence. (B) Ligation of adapters to fragmented DNA generates unique 12 bp tags on each end (α and β). PCR amplification of the two strands produces two related, but distinct products. (C) Sequence reads sharing unique α and β tags are grouped into families of α-β or β-α orientation. Mutations are of three different types: sequencing mistakes or late arising PCR errors (blue or purple spots); first round PCR errors (brown spots); true mutations (green spots). Comparing SSCSs from the paired families generates a DCS, which eliminates all but true mutations.

Duplex Sequencing relies on the concept of molecular tagging and the fact that the two strands of DNA contain complementary information. Fragmented duplex DNA is tagged with adapters bearing a random, yet complementary, double-stranded nucleotide sequence (Fig. 1A). Following ligation, the individually labeled strands are PCR amplified, thus creating sequence “families” that share a common tag sequence, derived from each of the two single parental strands (Fig. 1B). After sequencing, members of each duplicate family are grouped by tag, and a consensus sequence is calculated for each family, creating a single strand consensus sequence (SSCS) (Fig. 1C). This step eliminates random sequencing or PCR errors that occur during library amplification; however, the single-stranded consensus process does not filter out artifactual mutations that are the consequence of first round PCR errors, such as those commonly caused by DNA damage. To remove this latter type of error, the complementary SSCS families derived from the two single-stranded halves of the original DNA duplex are compared to each other (Fig. 1C). The base identity at each position in a read is kept in the final consensus only if the two strands match perfectly at that position. Upon remapping of these duplex consensus sequence (DCS) reads back to the reference genome, any deviations from the reference sequence are considered true mutations. The frequency of mutations in a sampled population of mtDNA is calculated as the number of DCS mutant molecules divided by the number of DCS wild-type molecules observed at any given genomic position.

### Point mutations accumulate with aging in human mtDNA

**Figure 2:**
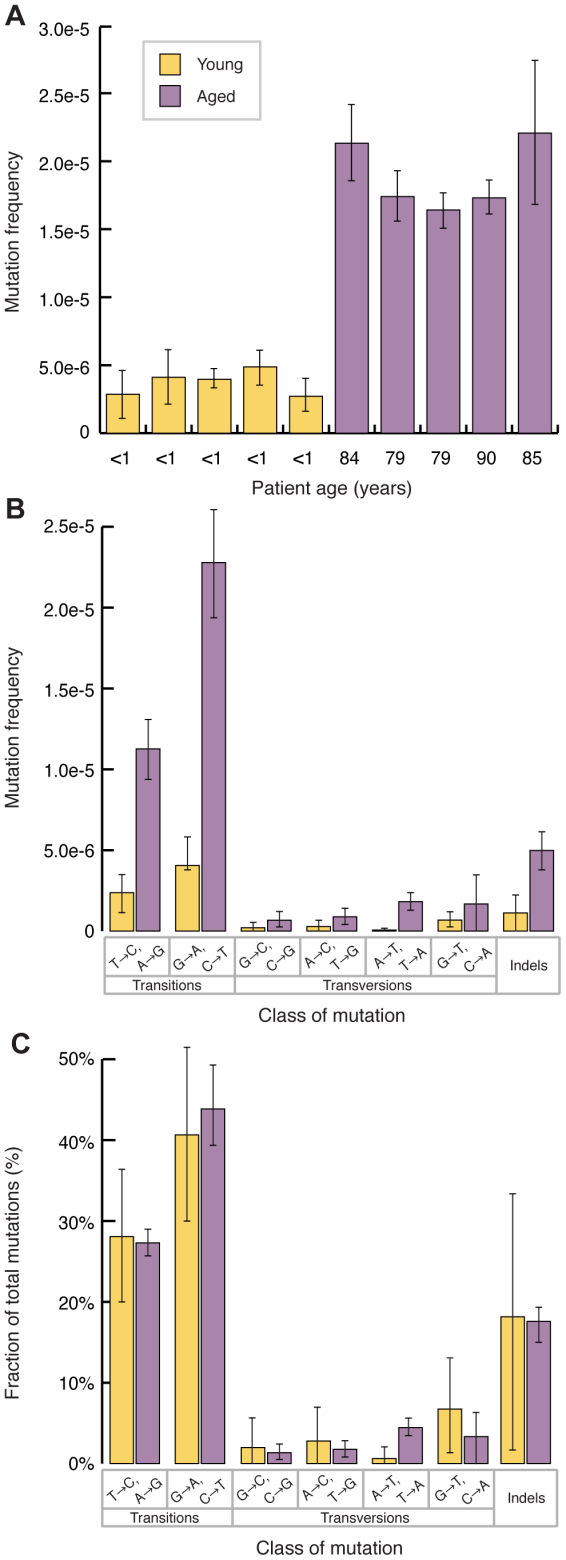
Mitochondrial point mutations increase with age and are biased to transitions. (A) mtDNA point mutations burden is higher in older individuals (*purple*) than young individuals (*yellow*) (p<10^−4^, two-tailed t-test). Error bars represent the 95% confidence interval for each sample (Wilson Score interval) (B) The mutation spectra of both young (*yellow*) and aged (*purple*) individuals shows an excess of transitions, relative to transversions. Frequencies were calculated by dividing the number of mutations of each type by the number of times the wild-type base of each mutation type was sequenced. Indels were calculated independently as events per total number of bases sequenced. Error bars represent one standard deviation. (C) The mutation spectra, reported as the relative proportion of the different mutation types, do not change with age. Error bars represent one standard deviation. Significance was tested using the two-tailed t-test.

Point mutations in mtDNA could be the result of maternal inheritance or a *de novo* mutation event. Maternally inherited mutations or mutations arising during early embryonic development are more likely to be clonal (i.e. the same mutation being present at the same location in most or all mtDNA molecules). Therefore, in order to quantify the frequency of *de novo* events, we used a clonality cutoff that excluded any positions with variants occurring at a frequency of >1%, and scored each type of mutation only once at each position of the genome. Based on these criteria, the mtDNA from aged individuals show a highly significant ∼5-fold increase in mutational frequency, relative to those obtained from young individuals (Young: 3.7±0.9×10^−6^ vs. Aged: 1.9±0.2×10^−5^, p<10^−4^, two-sample t-test) (Fig. 2A). These mutation frequencies are between one and two orders of magnitude lower than the previously reported values for both young and old individuals using PCR-based methods or conventional next-generation sequencing [24], [33], [34]. This discordance likely stems from artifactual scoring of mutations by these latter methods due to misinsertion of incorrect bases at sites of damage in template DNA during the PCR steps. Duplex Sequencing, in contrast, is unaffected by DNA damage [32].

Inspection of the mutation spectra for both the young and old samples reveals that all samples are significantly biased towards transitions (Fig. 2B). Specifically, the most common mutation type, G→A/C→T, is consistent with either misincorporation by DNA polymerase γ or deamination of cytosine to form uracil, as being the largest mutagenic drivers in mtDNA [35], [36]. The second most common mutation type, T→C/A→G, is consistent with either deamination of adenosine to inosine or a T-dGTP mispairing, the primary base misinsertion mistake made by DNA polymerase γ [37], [38], [39], [40]. Plotting the frequency of each type of mutation as a proportion of total mutations (Fig. 2C) reveals that the relative abundance of each mutation type is the same in young and old, suggesting that the mutagenic pressures that result in the observed spectra are constant throughout the human lifespan.

Surprisingly, comparison of the mutation spectra of the young and old samples reveals a notable absence of the mutational signature of oxidative damage. A number of studies have shown that oxidative damage to DNA accumulates in both the nuclear and mitochondrial genomes as a function of age, as well as several age-associated pathologies [17], [18], [19], . The most frequent alteration produced by oxidative damage is 8-oxo-dG, which, when copied during replication or repair, results in dA substitutions, yielding G→T/C→A transversions [42]. A number of theories of aging invoke ROS-mediated damage to mtDNA as being a major driver of the aging phenotype (reviewed in [43] and [44]). A key prediction for these theories is that the frequency of G→T/C→A mutations would be expected to increase with time. We failed to find either a preponderance of G→T/C→A substitutions or a proportionally greater increase with age in this type of mutation relative to other types, despite a span of >80 years between our sequenced sample groups (Fig. 2C).

### Deleterious mutations increase with age

Our data indicate that point mutations increase with age and that these mutations are inconsistent with oxidative damage being a primary driver of mutagenesis; we next assessed whether these mutations lead to alterations in the protein coding sequence. We find that in the aged samples, 78.3% of mutations are non-synonymous. The incidence of non-synonymous mutations is close to the expected value of 75.7% for mtDNA that would occur if non-synonymous and synonymous mutations occur randomly. In contrast, only 62.9% of mutations are non-synonymous in the young samples. The reduced mutation load observed in the young samples is consistent with that negative intergenerational selection against such mutations and that this selection is relieved during development and could play a role in the aging phenotype.

However, the existence of a high load of non-synonymous mutations does not necessarily mean that the coding changes lead to functional protein alteration. To examine this possibility, we compared the predicted “pathogenicity” of all non-synonymous mutations in both the young and aged samples using MutPred [45], a software package that calculates the likelihood of a mutation being deleterious based on a number of criteria, including protein structure, the presence of functional protein motifs, evolutionary conservation, and amino acid composition bias. A score between zero and one is assigned to each mutation, with a higher score denoting a higher likelihood of being deleterious. Based on this analysis (Fig. S1), the predicted pathogenicity of mutations, indeed, increases with age (p<0.02, Wilcoxon Rank Sum analysis), suggesting that mutations acquired during aging may have functional consequences for the electron transport chain. A similar increase in predicted deleterious mutations was also observed using the SWIFT software package (data not shown). The increase in predicted pathogenicity is consistent with mutations causing coding changes occurring randomly and argues against a mechanism by which point mutations are selected against by the cell. Similar finding in clonally expanded mutations were recently reported in colon tissue show a similar increase in predicted pathogenic mutation in mtDNA [46].

### The D-loop of mtDNA exhibits an elevated mutation burden but is not a mutagenic ‘hotspot’

The mitochondrial genome can be divided into three different regions: 1) protein coding genes, 2) RNA coding genes (consisting of both rRNA and tRNA), and 3) non-coding/regulatory regions including the origin of replication known as the D-loop. Phylogenetic analysis of both human and other mammalian lineages has shown that population level single nucleotide variants (SNVs) tend to cluster in a number of ‘hotspots’ in the mitochondrial genome, most notably in Hypervariable Regions I and II of the D-loop [47], [48], [49]. We sought to determine if the distribution of non-clonal mutations within the mtDNA of individuals exhibited a uniform distribution or if certain regions of the genome similarly show variations in mutation frequency. Comparison of the mutation frequencies of the RNA coding genes to the protein coding genes yielded no significant differences in either the young or old samples (p = 0.15, two-tailed t-test).

**Figure 3:**
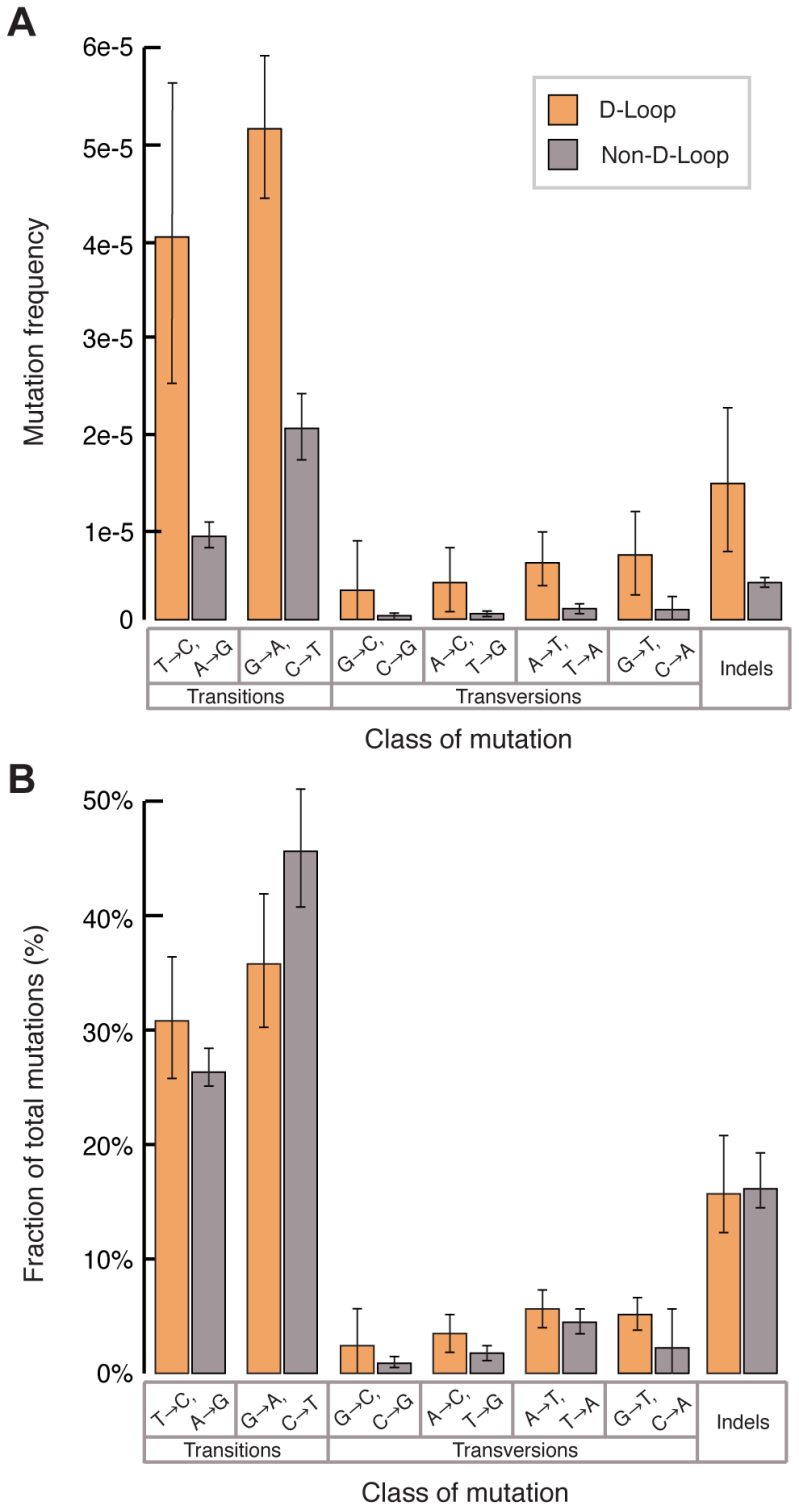
The D-loop has an elevated mutation burden but its mutation spectrum is similar to the remainder of the mitochondrial genome. (A) The D-loop (*orange*) exhibits a higher aggregate mutation burden than the rest of the genome (*grey*). Frequencies were calculated by dividing the number of mutations of each type by the number of times the wild-type base of each mutation type was sequenced. Indels were calculated independently as events per total number of bases sequenced. Error bars represent one standard deviation. (B) The relative fraction of mutations exhibits no difference between the D-loop (*orange*) and non-D-loop (*grey*) portions of the genome, suggesting a similar underlying mutagenic process. Error bars represent one standard deviation.

In contrast, we observed a significant increase in mutation frequency of the D-loop (bp 16024-576) relative to the coding regions (bp 577–16023) in both young (D-loop: 1.5±0.6×10^−5^ vs. coding region: 2.9±0.7×10^−6^, p<0.01, two-tailed t-test) and aged (D-loop: 5.7±1.5×10^−5^ vs. coding region: 1.65±0.2×10^−5^, p<0.01, two-tailed t-test) samples, suggesting that the D-loop is a mutagenic hotspot. However comparing the relative increase in the mutation frequency of the D-loop between the young and old sample groups (3.8±1.6-fold increase) to the relative increase seen between the two sample groups in the non-D-loop regions (5.6±2.0 fold increase) shows no difference. This finding is inconsistent with the idea that the D-loop accumulates significantly more mutations during aging than the rest of the mitochondrial genome. Spectrum analysis shows a similar predominance of transition mutations in both the D-loop and coding regions of the genome (Fig. 3A), with no significant difference in the relative abundance of the different mutation types (Fig. 3B). Taken together, our data suggest that the mutagenic processes of mtDNA are largely uniform across the genome.

### Mutations accumulate asymmetrically on the two strands of mtDNA

The human mitochondrial genome has a significant bias in the cytosine/guanine composition between the two strands. Specifically, the light strand (L-strand), which is the coding strand for only nine genes, contains about three-fold more cytosine than guanine, whereas the heavy strand (H-strand) codes for the remaining 28 genes and has the opposite composition bias. Human population studies, as well as the comparative analysis of evolutionarily related species, have shown a bias towards the occurrence of G→A and T→C SNPs of the L-strand [50], [51], [52], [53]. These population-level compositional biases are hypothesized to be due to an asymmetric accumulation of mutations between the two strands of mtDNA in the germline; however, to date, the biases have not been observed at the sub-clonal/random level within individuals. To examine this, we compared the frequency of reciprocal mutations occurring on the L-strand (i.e. G→A on the L-strand vs. C→T mutations on the L-strand). By definition, mutations cause complementary sequence changes on both strands of a DNA molecule. Therefore, if a bias does not exist in the orientation of specific mutations towards a particular strand, then the frequency of reciprocal mutations on the same strand would be expected to be equal. Alternatively, the presence of a strand orientation bias would manifest itself in the form of a particular type of mutation occurring more frequently than its reciprocal mutation.

**Figure 4:**
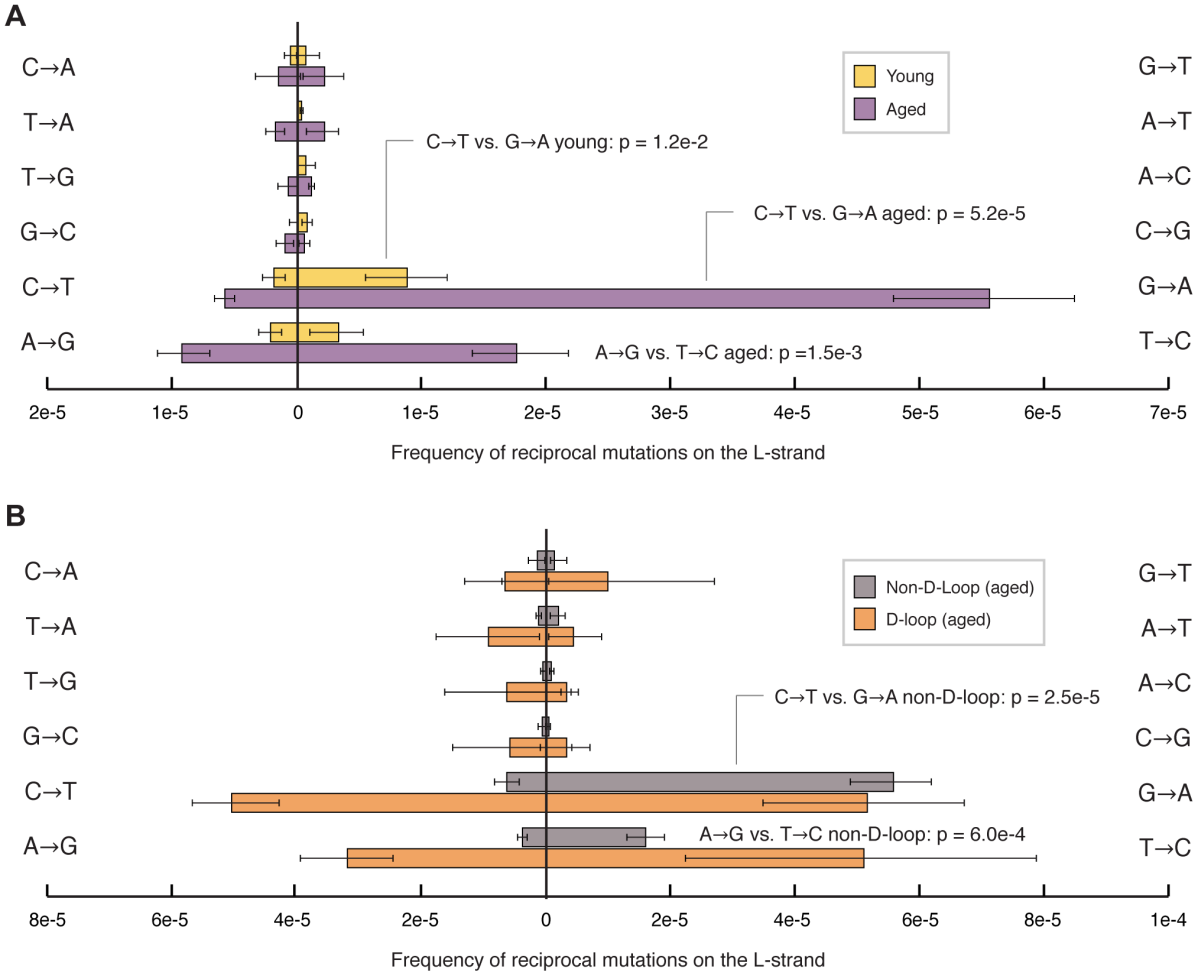
Transition mutations show a pronounced strand bias in their occurrence. (A) Comparison of frequencies of reciprocal mutation types on the L-strand for both young (*yellow*) and aged (*purple*) sample groups shows that young individuals exhibit a strand bias in the occurrence of G→A/C→T mutations. Aged samples exhibit an even stronger bias in both G→A/C→T and T→C/A→G mutations. No strand bias was observed for transversion mutations. (B) Comparison of frequencies of reciprocal mutation types on the L-strand from aged individuals shows no significant strand bias in the D-loop (bp 16,025-576) region (*grey*) of mtDNA, while the non-D-loop portion (bp 577–16,024) (*orange*) of the genome from the same aged samples exhibits a strong strand bias. Frequencies were calculated by dividing the number of mutations of each type by the number times the wild-type base of each mutation type was sequenced. Error bars represent one standard deviation. Significance was tested using the two-tailed t-test.

We find that the majority of the human mitochondrial genome shows a significant strand orientation bias in the occurrence of transitions, whereas transversions show no apparent asymmetry (Fig. 4A). Specifically, in young samples, G→A/C→T mutations are more likely to occur when the dG base is present on the L-strand and the dC base is in the H-strand, respectively. This pattern is even more pronounced in aged individuals, consistent with this bias being due to ongoing mutagenic process and not the result of maternal inheritance. In addition to the G→A/C→T bias, the aged samples also exhibit a strand orientation bias in the occurrence of T→C/A→G, where dT is more likely to be mutated to a dC when it is located on the L-strand than on the H-strand. Interestingly, this bias, which appears uniformly throughout most of the mtDNA, is uniquely absent in the D-loop region (Fig. 4B). Thus, both the spectrum and strand orientation asymmetry of somatic mtDNA mutation accumulation recapitulates what has been previously recognized in population studies.

## Discussion

The accumulation of somatic mutations in mtDNA has frequently been hypothesized to drive the aging process and its associated pathologies, including neurodegeneration, cancer, and atrophy (reviewed in [54]). The underlying mechanisms by which these mutations occur and accumulate have been the subject of intense study, but remain incompletely defined. One of the major limitations has been the lack of methodologies with sufficient sensitivity to detect rare mutations among a much larger population of wild-type molecules. We recently developed a robust next-generation sequencing methodology, termed Duplex Sequencing, which is able to detect a single point mutation among >10^7^ sequenced bases [32] and has now enabled us to precisely characterize the genome-wide frequency, spectrum, and distribution of somatic mtDNA mutations in aging human brain with unprecedented accuracy.

Our data show a significant increase in the load of point mutations as a function of human age, with absolute frequencies 10–100 fold lower than what has been typically reported in the literature using less sensitive assays. Recent work using the Random Mutation Capture assay has reported an age associated increase in mtDNA point mutation frequencies in mice and *Drosophila* that are on par with the values that we have determined here; however, these studies were limited to only a very small region of the genome [22] (Leo Pallanck-submitted). Of particular interest, despite a ∼1000-fold difference in lifespan, the increase in mutation load with age appears to be highly consistent among multiple species. This surprising finding suggests that the underlying mechanisms behind the age-dependent accumulation of point mutations in mtDNA are conserved between humans, flies, and mice and merit more detailed comparison.

Oxidative damage to DNA, most notably in the form of 8-oxo-dG, has long been believed to be a primary driver of mutagenesis in both nuclear and mitochondrial DNA [42], [55], [56]. However, our results do not support this hypothesis. In our data, the relative proportion of G→T/C→A mutations is quite low in the young samples examined and, importantly, does not show a disproportionate increase with age relative to other types of mutations. Other recent reports, which used less sensitive methods to detect intermediate frequency sub-clonal mutations, have similarly failed to detect this classic signature of oxidative damage to DNA. For instance, one conventional deep sequencing analysis of aged mice reported no significant burden of G→T/C→A transversions [57]. Even more surprising is the observation that a transgenic mouse strain deficient for both MutY and OGG1, which are the two primary enzymes responsible for repairing 8-oxo-dG, do not exhibit an increase in mtDNA mutations [58].

Comparison of the spectrum of our reported data (Fig. 2B) to that of the clonal SNV's in our data (i.e. mutations present at >90%), as well as those reported in the Mitomap database [59], reveals an identical bias towards transitions with a minimal number of G→T/C→A transversions (Fig. S2). Indeed, a similar propensity towards transitions has been noted in numerous animal phylogenies [60]. This consistency in mutational pattern suggests that the mutagenic processes that cause the accumulation of mutations in somatic tissue are also responsible for clonal population variants arising in the maternal germline.

In addition to 8-oxo-dG, ROS can also cause a number of other mutagenic lesions, including thymine glycol and deamination of cytidine and adenosine, all of which can induce transition mutations [37], [61], [62]. Our data clearly show an excess of transitions relative to transversions, which could be consistent with oxidatively induced deamination events becoming fixed as mutations. It is well established that ROS production and oxidative damage increase with age [17], [18], [19], [20]. Yet, if oxidative damage were the main driver for deamination events, this model would predict that the relative proportion of transitions should be disproportionately higher in aged individuals, which is not the case with our data. While we cannot conclusively rule out a role for ROS in inducing transition mutations, the excess of transitions could additionally be explained by spontaneous deamination of either cytidine or adenosine, especially in single-stranded replication intermediates, or base misincorporation events by DNA polymerase γ during genome replication, which has a known propensity for transition mutations [36], [39], [40]. Overall, the absence of key mutagenic signatures of oxidative damage argues against ROS being the major driver of mutagenesis in mtDNA in normal aging brain.

Several explanations may account for why we observed few 8-oxo-dG associated mutations in mtDNA despite an extensive literature showing that 8-oxo-dG levels increase with age. First, rapid DNA repair may remove 8-oxo-dG prior to genome replication and this repair capacity may increase with age [63], thereby keeping 8-oxo-dG mutagenesis to a minimum. Secondly, mitochondrial quality control pathways may simply eliminate mitochondria with damaged mtDNA. Consistent with this idea is the observation that oxidatively damaged mtDNA rapidly disappears from cells treated with H_2_O_2_ [64]. In addition, the cellular levels of parkin, a major component of the quality control pathway involved in mitochondrial turnover, increase under conditions of high oxidative stress [65]. Finally, DNA polymerase γ itself may actively discriminate against incorporating 8-oxo-dG [66]. Regardless of the mechanism, our data suggest that cells have evolved one or more strategies to effectively deal with the challenge of replicating mtDNA in the highly damaging environment of the mitochondria.

We find a striking excess of mutations in the D-loop in young individuals; however, D-loop mutations accumulate during aging at the same rate as other parts of the mitochondrial genome, consistent with the D-loop not being inherently error prone. Instead, our data suggest that young individuals are born with a higher aggregate mutation burden in the D-loop relative to the rest of the genome, thus preserving the D-loop's disproportionate mutation load as mutations accumulate during life. The higher proportion of low-level/random mutations in the D-loop also offers a likely explanation as to why population level SNVs tend to cluster in a number of ‘hotspots’ in the D-loop.

The disproportionate mutation load of the D-loop at birth provides a likely explanation as to why the D-loop has long been considered prone to mutagenesis. Due to the D-loop's higher aggregate burden, the previously used assays were only sensitive enough to detect mutations located in the D-loop. Thus, even though point mutations increase uniformly throughout mtDNA with age, the mutation load in the non-D-loop portions of the genome would likely be below the sensitivity of the previously available assays, thus giving the appearance that the D-loop's mutations increased with age.

Human population studies have previously identified a bias in the occurrence of G→A and T→C SNPs of the L-strand, as has comparison of human mtDNA sequences with those of evolutionary related species [50], [51], [52]. This imbalance has been hypothesized to be the result of preferential deamination of cytidine and adenosine on the H-strand; however, because this strand orientation bias has only been previously observed at the clonal population scale, the influence of natural selection could not be discounted [50], [52]. Our data demonstrate that this bias originates from mutagenesis of mtDNA through a process that is continuous throughout life; however, the source of this bias is unclear. One possibility is that this bias arises during mtDNA replication. Replication of mtDNA is thought to occur in an asymmetric manner [67], [68]. In this model, replication of the H-strand (G-rich strand of mtDNA) begins at the H-strand origin, using the L-strand (C-rich strand of mtDNA) as its template. During this time, the parental H-strand remains in a single-stranded state until the synthesis of the L-strand is initiated at the L-strand origin. Several studies previously documenting a strand orientation bias of clonal SNVs at the population level hypothesized that the bias is due to an increased rate of spontaneous hydrolytic deamination of cytidine and adenosine on the H-strand spending a greater amount of time in an unprotected single-stranded state during replication [50], [51], [52], [69]. Indeed, there is *in vitro* evidence that replication past a deaminated dC (i.e. dU) by DNA polymerase γ is responsible for the observed asymmetry. Zheng *et al.* used DNA polymerase γ to amplify small regions of homoduplex mtDNA via PCR and observed the emergence of an excess of G→A/C→T mutations in these reactions [40]. Their experimental conditions involved extended heating of the DNA, which is known to increase the rate of deamination of single-stranded DNA. While our results do not, on their own, provide direct proof that an asymmetry in deamination of single-stranded replication intermediates is responsible for the observed inter-strand mutational skew, our data are consistent the findings and conclusions in Zheng *et al.*, thus providing strong support for this model [40]. Furthermore, the observation that a similar strand bias exists in the absence of intergenerational selection argues that it is due to an ongoing mutagenic process within the cell and that the bias observed at the population level is likely due to the same process.

In summary, Duplex Sequencing is a powerful technology for high-sensitivity study of mtDNA mutagenesis that has enabled us to uncover a number of mutagenic patterns and biases in human brain tissue that have not been previously observed at the somatic level. Taken together, these mutation patterns argue against oxidative DNA damage being a major driver of aging and suggest that replication errors by DNA polymerase γ and/or spontaneous base hydrolysis are responsible for the bulk of point mutations that accumulate in mtDNA. In light of this finding, the central role for oxidative damage to mtDNA in theories of human aging and disease merits re-evaluation.

## Materials and Methods

### Tissues and DNA isolation

Mitochondria were isolated from the pre-frontal cortex of either young (<1 yr) or old (>75 yr) brain tissue with no known brain pathologies (Table S1). Approximately 500–800 mg of brain tissue was sub-divided into smaller 100–150 mg pieces, with the mitochondria from each piece purified independently from the other pieces of the same tissue sample. Each tissue fraction was dounced in 5 mL of homogenization buffer (0.32 M sucrose, 1 mM EDTA, 10 mM Tris-HCl, pH 7.8) with no more than five strokes, as excessive douncing increases the contamination of nuclear DNA. The homogenate was centrifuged at 1000 g for 20 min at 4°C. The supernatant was transferred to a new centrifuge tube and centrifuged at 1000 g for 10 min at 4°C. Samples that exhibited a pellet at the bottom of the tube after the second centrifugation step were centrifuged a third time using the same condition. The supernatant was removed and centrifuged at 13,000 g for 30 min at 4°C to give a crude mitochondrial pellet. Each crude pellet was resuspended in Mito-DNase buffer (0.3 M sucrose, 10 mM MgCl_2_, 0.15% BSA (w/v), 20 mM Tris-HCl, pH 7.5) and DNase added to a concentration of 0.01 mg/mL and incubated at 37°C for 1.5 hrs. This step helps remove contaminating nuclear DNA while not affecting mtDNA protected within intact mitochondria. Mitochondria were then re-pelleted at 13,000 g for 30 min and the supernatant discarded. The mitochondrial pellets were washed two times by resuspending the pellets in 1 mL of Mito-DNase buffer followed by centrifugation at 13,000 g for 30 min. Finally, the enriched mitochondrial pellets were resuspended in 500 µL Lysis Buffer (150 mM NaCl, 20 mM EDTA, 1% SDS (w/v), 10 mM Tris-HCl, pH 7.8, 0.2 mg/mL Proteinase K, 0.01 mg/mL RNase) and incubated at 56°C for 1 hr. The mtDNA was extracted using a standard phenol∶chloroform approach. The relative purity of each mtDNA prep from nuclear DNA was determined by qPCR using the following primers sets: nDNA: *fwd* 5′- TTGCCAGACCATGGGATTGTCTCA, *rev* 5′-TTCCTACCGAACGAGGACTCCAAA; mtDNA: *fwd* 5′-ACAGTTTATGTAGCTTACCTC, *re*v 5′-TTGCTGCGTGCTTGATGCTTG. DNA fractions from the same tissue samples exhibiting a ΔC(t) ≥17.5 cycles (corresponding to ∼50% mtDNA by mass) were pooled and used for library preparation and sequencing. Typical yields were between 100–500 ng of highly pure mtDNA.

### Sequencing

We carried out synthesis and preparation of the Duplex Tag-labeled adapters and sequencing library preparation as previously described with the following minor mtDNA specific modifications [32]: 1) 100–500 ng of mtDNA was sheared using the Covaris AFA system with a duty cycle of 10%, intensity of 5, cycles/burst 100, time 20 seconds×5, temperature of 4°C; 2) prior to adapter ligation, the DNA was quantified and a 40∶1 molar excess of adapters was used for the ligation step; 3) after adapter ligation and clean up, the library was re-quantified and ∼37 fmol of product was amplified by PCR for a total of 20 cycles. The resulting libraries were subjected to sequencing on an Illumina HiSeq 2000/2500 platform using 101 bp, paired-end reads.

#### Data analysis

The overall sequencing pipeline has been previously described [32]. Briefly, all raw sequencing reads are filtered based on the location of an expected fixed sequence within each read, as well as the fact that each position of the 12 nucleotide random Duplex Tag sequence can only contain one of the four canonical bases. Any reads not conforming to these criteria are discarded. The random tag sequences from each read pair are computationally concatenated into a 24-nt random sequence and appended to the header of each read-pair. In order to remove potential artifactual mutations arising from the end-repair and ligation reactions, each read was trimmed by four additional bases. The reads were then aligned against build 19 of the human genome using the Burrows-Wheeler Aligner (BWA) [70]. Reads not aligning to the human mitochondrial genome were filtered out. A consensus sequence for each tag family (e.g. reads sharing identical tag sequences) was computationally determined using software written in-house. The consensus for any position in a read was considered undefined if the position was represented by fewer than three instances in the family or if less than 70% of the sequences at that position in the read were in agreement. The SSCS reads were then realigned to the revised mitochondrial Cambridge Reference Sequence using BWA. After filtering for unmapped reads, DCS reads were constructed by pairing SSCS with their respective strand-mates by grouping the 24 nucleotide tag in read 1 with the appropriate 24 nucleotide tag in read 2 and the sequence identity at each position of the two reads were compared to one another. The sequence information was kept only if the base identity of both reads were identical. Next, to remove alignment artifacts common at the ends of reads, five nucleotides from each end of the duplex reads were soft-clipped using the Genome Analysis Toolkit. Finally, all reads that mapped to the same genomic coordinates and contained tags that differed in their sequence by less than three nucleotides were removed from the data set. Reads found to harbor a mutation were then realigned against the entire human genome using BLAST. Reads that had a lower e-score for a region of the nuclear genome than the mtDNA were removed in order to eliminate any potential contribution from nuclear mitochondrial pseudogenes. Notably, on post-analysis review, no correlation existed between sample mutation frequency and degree of nuclear genome contamination, which would be expected if mutation data were confounded by contaminating clonal mutations in nuclear pseudogenes (Fig. S3). Scripts to calculate mutation frequencies and locations were written in-house and are provided as supplemental files. Due to the high redundancy of the sequencing data in Duplex Sequencing, relatively few “genome equivalents” are sequenced (∼5,000 “genomes”/sample), thus the ability to detect sub-clonal, multi-kilobase, deletions and rearrangements is limited and was, therefore, not evaluated in this study.

### Ethics statement

This study involved the use of human brain tissue obtained from autopsy. The tissues were collected at University of Washington under the direction of the Alzheimer's Research Center and Seattle Children's hospital under their approved IRB protocols. Our research did not fall under human subjects requirements due to the samples being anonymous.

## Supporting Information

Figure S1 Non-synonymous mutations are predicted to be more deleterious with age. All non-synonymous, non-clonal (i.e. occurring at <1% clonality) mutations were scored for pathogenicity using MutPred. Young (*gray*) samples had a significantly lower pathogenicity score than the aged samples (*light blue*) (p<0.02, Wilcoxon Rank Sum analysis).
(TIF)

Figure S2 Population level SNPs, homoplasmic variants, and non-clonal mutations all show a similar mutation spectra. Mitomap.org data (*light blue*) were downloaded from http://www.mitomap.org/bin/view.pl/MITOMAP/PolymorphismsCoding and http://www.mitomap.org/bin/view.pl/MITOMAP/PolymorphismsControl. Insertion/deletion mutations were ignored and only point mutations were tabulated. Homoplasmic variants from our data set (*dark blue*) are defined as variants occurring in >90% of mapped reads. We detected a total of 134 unique homoplasmic variants among all young and aged samples. Data for the Non-clonal variants (*green*) are derived from the aged sample data in Figure 2B.
(TIF)

Figure S3 Nuclear DNA contamination does not affect observed mtDNA mutation frequency. Analysis was performed on reads only mapping to either the nuclear or mitochondrial genome. Percentage of mtDNA was calculated by dividing the number of reads mapping to the mtDNA by the total number of mapped reads. Linear regression is non-significant (R^2 = ^0.15).
(TIF)

Table S1 Sample information and sequencing statistics.
(DOCX)

Text S1 Scripts used for data processing and analysis.
(ZIP)
