# Supplementary figures and images for: Ultra-Sensitive Sequencing Reveals an Age-Related Increase in Somatic Mitochondrial Mutations That Are Inconsistent with Oxidative Damage
Source: PLoS Genet. 2013 Sep 26;9(9):e1003794. doi: 10.1371/journal.pgen.1003794 (PMC3784509; doi:10.1371/journal.pgen.1003794)

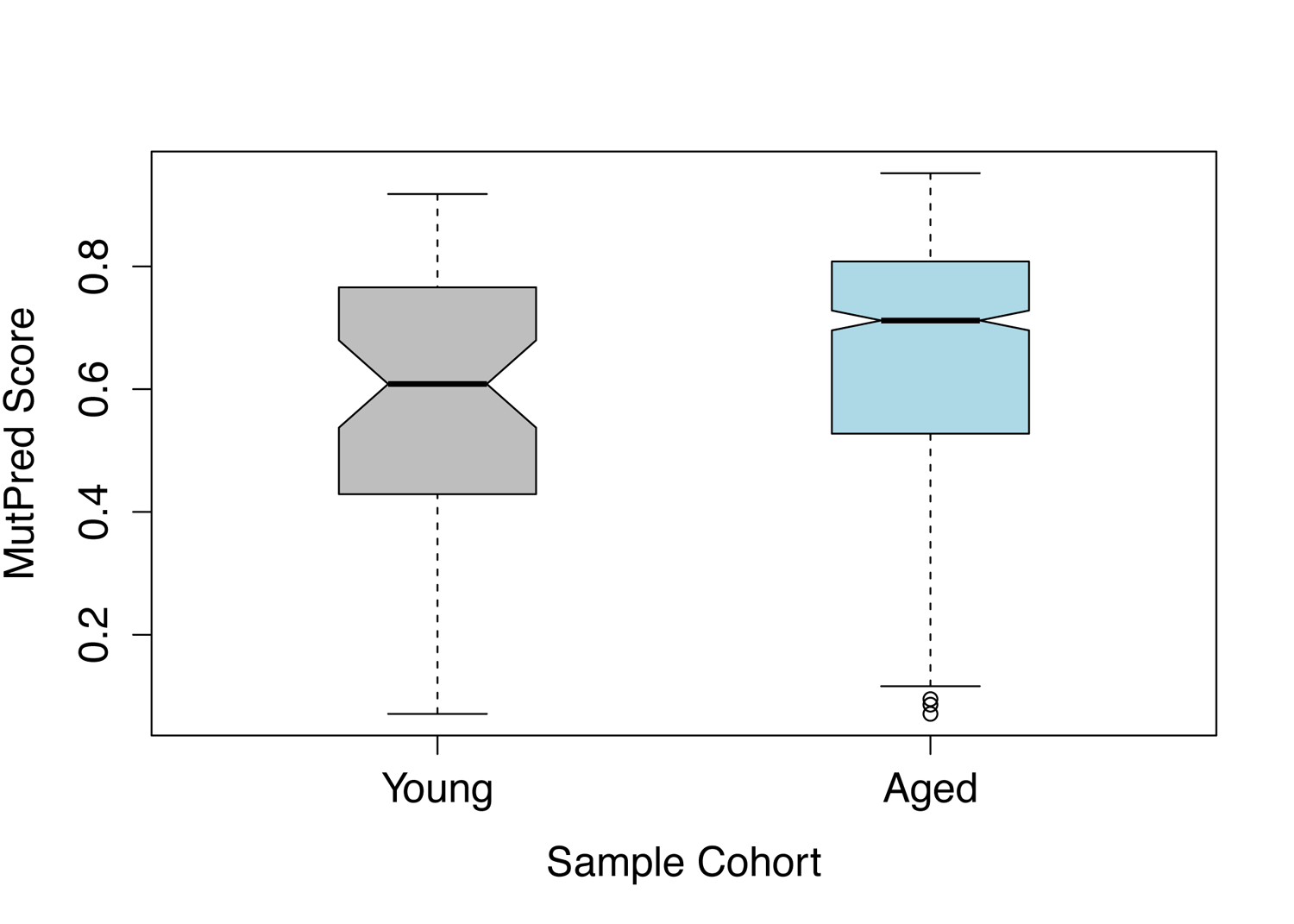

Supplement: Figure S1 — Non-synonymous mutations are predicted to be more deleterious with age. All non-synonymous, non-clonal (i.e. occurring at <1% clonality) mutations were scored for pathogenicity using MutPred. Young (gray) samples had a significantly lower pathogenicity score than the aged samples (light blue) (p<0.02, Wilcoxon Rank Sum analysis). (TIF) [file pgen.1003794.s001.tif]

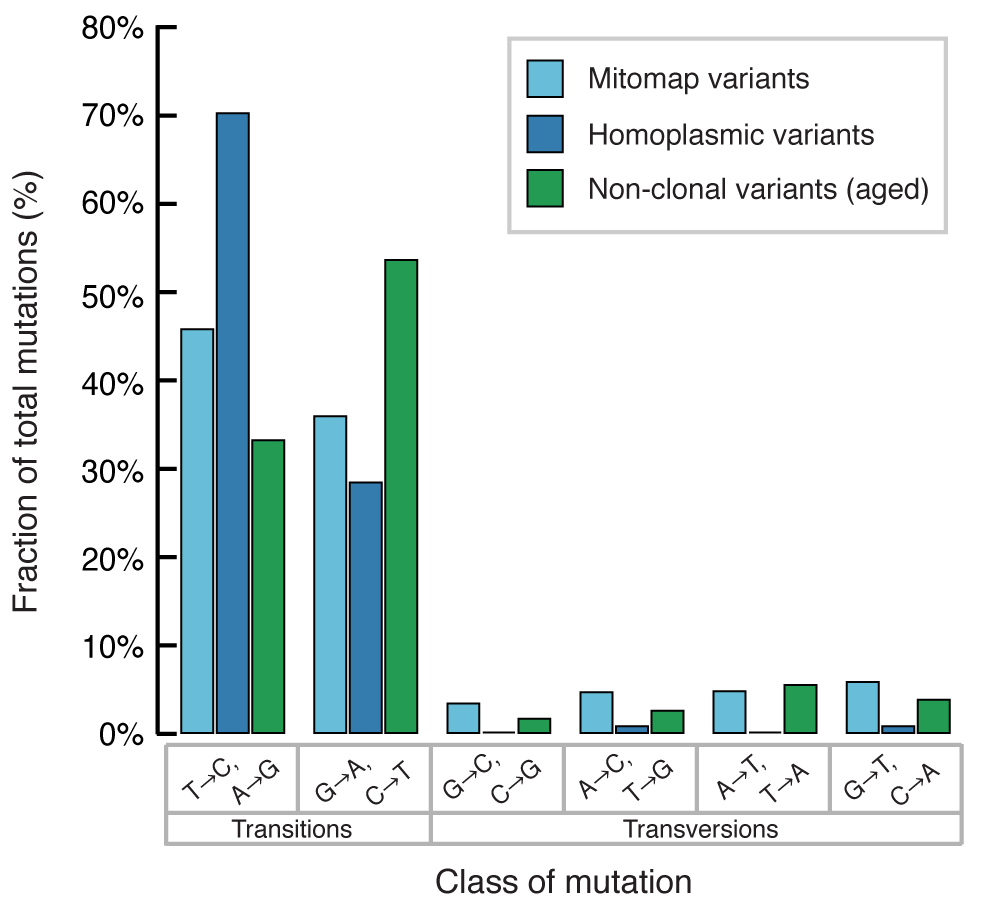

Supplement: Figure S2 — Population level SNPs, homoplasmic variants, and non-clonal mutations all show a similar mutation spectra. Mitomap.org data (light blue) were downloaded from http://www.mitomap.org/bin/view.pl/MITOMAP/PolymorphismsCoding and http://www.mitomap.org/bin/view.pl/MITOMAP/PolymorphismsControl. Insertion/deletion mutations were ignored and only point mutations were tabulated. Homoplasmic variants from our data set (dark blue) are defined as variants occurring in >90% of mapped reads. We detected a total of 134 unique homoplasmic variants among all young and aged samples. Data for the Non-clonal variants (green) are derived from the aged sample data in Figure 2B. (TIF) [file pgen.1003794.s002.tif]

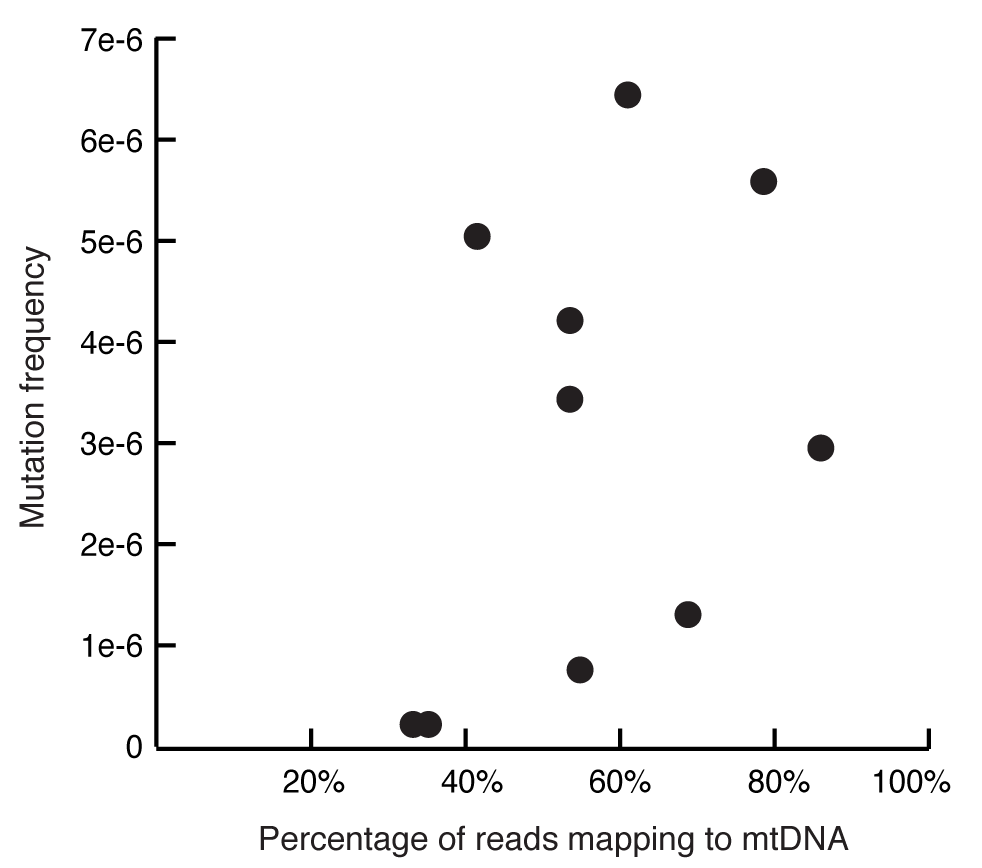

Supplement: Figure S3 — Nuclear DNA contamination does not affect observed mtDNA mutation frequency. Analysis was performed on reads only mapping to either the nuclear or mitochondrial genome. Percentage of mtDNA was calculated by dividing the number of reads mapping to the mtDNA by the total number of mapped reads. Linear regression is non-significant (R2 = 0.15). (TIF) [file pgen.1003794.s003.tif]
