# Supplementary material for: Ultra-Sensitive Sequencing Reveals an Age-Related Increase in Somatic Mitochondrial Mutations That Are Inconsistent with Oxidative Damage
Source: PLoS Genet. 2013 Sep 26;9(9):e1003794. doi: 10.1371/journal.pgen.1003794 (PMC3784509; doi:10.1371/journal.pgen.1003794)
Supplement: Table S1 — Sample information and sequencing statistics. (DOCX) [file pgen.1003794.s004.docx]

Supplemental Table 1: Sample Information and Sequencing Statistics

| **Sample ID** | **Age (years)** | **Avg. Coverage** | **Total Sequenced Nucleotides** | **Purity**  **(% reads mapping to mtDNA)** | | **Point Mutation Frequency** | |
| --- | --- | --- | --- | --- | --- | --- | --- |
| **Young Samples** |  |  |  |  | |  | |
| SC1 | <1 | 233 | 3849720 | 35% | | 2.86x10^-6^ | |
| SC2 | <1 | 251 | 4141158 | 33% | | 4.11x10^-6^ | |
| SC3 | <1 | 1866 | 30786977 | 69% | | 3.96x10^-6^ | |
| SC4 | <1 | 697 | 11498938 | 55% | | 4.87x10^-6^ | |
| SC5 | <1 | 447 | 7369369 | 86% | | 2.71x10^-6^ | |
|  |  | |  |  |  | |  |
|  |  | |  |  |  | |  |
| **Aged Samples** |  | |  |  |  | |  |
| UWA498 | 84 | 657 | 10832804 | 79% | | 2.13x10^-5^ | |
| UWA533 | 79 | 1188 | 19599434 | 54% | | 1.74x10^-5^ | |
| UWA549 | 79 | 2252 | 37165412 | 55% | | 1.64x10^-5^ | |
| UWA679 | 90 | 2753 | 45418203 | 42% | | 1.73x10^-5^ | |
| UWA738 | 85 | 187 | 3081423 | 61% | | 2.21x10^-5^ | |
